# Turning a Corner in ME/CFS Research

**DOI:** 10.3390/medicina57101012

**Published:** 2021-09-25

**Authors:** Derek F. H. Pheby, Kenneth J. Friedman, Modra Murovska, Pawel Zalewski

**Affiliations:** 1 Society and Health, Buckinghamshire New University, High Wycombe HP11 2JZ, UK; 2 School of Osteopathic Medicine, Rowan University, Stratford, NJ 08080, USA; kenneth.j.friedman@gmail.com; 3 Institute of Microbiology and Virology, Riga Stradiņš University, LV-1067 Riga, Latvia; Modra.Murovska@rsu.lv; 4 Department of Exercise Physiology and Functional Anatomy, Ludwik Rydygier Collegium Medicum in Bydgoszcz, Nicolaus Copernicus University in Torun, Swietojanska 20, 85-077 Bydgoszcz, Poland; p.zalewski@cm.umk.pl

**Keywords:** ME/CFS, myalgic encephalomyelitis, chronic fatigue syndrome, knowledge and understanding, quality of life, guideline, clinical care

## Abstract

This collection of research papers addresses fundamental questions concerning the nature of myalgic encephalomyelitis/ chronic fatigue syndrome (ME/CFS), the problem of disbelief and lack of knowledge and understanding of the condition among many doctors and the origins of this problem, and its impact on patients and their families. We report briefly the growing knowledge of the underlying pathological processes in ME/CFS, and the development of new organizations, including Doctors with ME, the US ME/CFS Clinical Coalition and EUROMENE, to address aspects of the challenges posed by the illness. We discuss the implications of COVID-19, which has much in common with ME/CFS, with much overlap of symptoms, and propose a new taxonomic category, which we are terming post-active phase of infection syndromes (PAPIS) to include both. This collection of papers includes a number of papers reporting similar serious impacts on the quality of life of patients and their families in various European countries. The advice of EUROMENE experts on diagnosis and management is included in the collection. We report this in light of guidance from other parts of the world, including the USA and Australia, and in the context of current difficulties in the UK over the promulgation of a revised guideline from the National Institute for Health and Care Excellence (NICE). We also consider evidence on the cost-effectiveness of interventions for ME/CFS, and on the difficulties of determining the costs of care when a high proportion of people with ME/CFS are never diagnosed as such. The Special Issue includes a paper which is a reminder of the importance of a person-centred approach to care by reviewing mind–body interventions. Finally, another paper reviews the scope for prevention in minimizing the population burden of ME/CFS, and concludes that secondary prevention, through early detection and diagnosis, could be of value.

Myalgic encephalomyelitis/chronic fatigue syndrome (ME/CFS) is a relatively common and often misunderstood illness, which causes considerable distress to patients and their families, not least because of frequently encountered unhelpful and judgmental attitudes on the part of healthcare professionals. It also has considerable economic implications, and a focus group based in Ireland found that ME/CFS patients incurred wide-ranging costs, as well as wider societal costs including health care costs, lost productivity, and impacts on informal carers [1].

This collection of research papers concerning ME/CFS addresses some important issues. One such issue goes to the very heart of the debate within the medical profession about the very nature of the disease. Many doctors refuse to accept that ME/CFS is a genuine clinical entity, and ascribe it instead to a variety of psychiatric diagnoses. A major cause of doctors’ disbelief in ME/CFS is the 1970 paper by McEvedy and Beard in the BMJ, which determined that the 1958 Royal Free epidemic of ME/CFS was ‘epidemic hysteria’ [2]. However, the report in this collection by Underhill and Baillod [3], based on a focus group and interviews with survivors of the 1958 Royal Free epidemic, corroborates a paper published last year which invalidated the McEvedy/Beard paper on the basis of their having identified mathematical errors in the original publication [4]. The weaknesses in the McEvedy/Beard paper spotlighted by this triangulation of a mathematical and an historical approach should be sufficient to consign the hysteria hypothesis to the dustbin of history, where it belongs.

That ME/CFS is a clinical entity is underlined by an increasing corpus of knowledge of the pathological processes underlying ME/CFS. ME/CFS is a multi-system disorder, with dysregulation of the HPA axis and of metabolism of the central nervous system and of body systems generally. The range of abnormal responses includes alteration of autonomic nervous system function, in particular sensitization of the sympathetic nervous system, with lasting adaptations in energy metabolism and the immune response, orthostatic intolerance with reduction in cerebral blood flow on tilt testing, variations in cortisol levels associated with increased fatigue, disorganized circadian rhythms, increased immune system activation, as shown for example by increased pro-inflammatory cytokines and prolonged inflammatory responses, alterations in muscle anaerobic threshold, abnormal recovery after activity with post-exertional malaise, central sensitization, and changes in grey and white matter in the brain [5]. ME/CFS is neither a rare nor an orphan disease, with perhaps as many as 2.5 million people suffering from it in the USA, and there is now new evidence of underlying anatomical, physiological and electrical abnormalities in the brain, of chronic activation of the immune system, including autoantibodies directed at the central and autonomic nervous systems, of impaired energy metabolism with associated oxidative stress, of dysregulation of the autonomic nervous system, and of abnormalities of the gut microbiome [6]. Some blood test results differed in ME/CFS patients from those of healthy controls. In particular, creatine kinase and creatinine were reduced in ME/CFS patients [7]. Metabolic dysfunction, resulting from abnormal immune responses, has been identified as possibly underlying the major symptoms of ME/CFS [8]. Evidence for an infection-triggered autoimmune process involving dysfunctional ß2-adrenoreceptor antibodies has been published, which may indicate a therapeutic role for immunoadsorption therapy. The latter has shown promising results in a pilot study [9].

The persistence of dismissive attitudes among doctors is a consequence of inadequate teaching about ME/CFS, both in medical schools to undergraduates and at the postgraduate level to physicians in practice. Four of the papers in this collection consider aspects of knowledge of ME/CFS among doctors. A literature review of GP knowledge and understanding of ME/CFS included in this collection of papers concluded that between a one-third and a half of GPs did not accept ME/CFS as a genuine clinical entity, and that a similar proportion of patients were dissatisfied with the primary medical care they had received [10]. These proportions applied geographically irrespective of location, and had changed little in recent decades. Furthermore, the study of perceptions of ME/CFS experts across Europe indicated that this situation was current throughout Europe [11]. An exploratory survey of UK medical schools by Muirhead et al. indicated that undergraduate teaching about ME/CFS was generally inadequate [12], while, at the postgraduate level, evidence is evinced here of considerable misconceptions about the nature of ME/CFS, its diagnosis and management among UK junior hospital doctors [13]. It should be appreciated that this failure to recognize ME/CFS as a genuine clinical entity is not merely an interesting academic dispute. For patients deprived of medical care as a result, and, worse, labelled at best as being malingerers and at worst of being seriously psychiatrically disturbed, it is nothing short of disastrous.

The literature review referred to above [10] found that ignorance and denial among doctors were paralleled by widespread dissatisfaction with their medical care among patients. Where doctors (or their family members) are themselves patients, they experience a double problem: not only are they as subject to diagnostic error as any other patient, but they are uniquely qualified to know when they are subject to such errors. In addition, they may find themselves experiencing unsympathetic responses from colleagues who have little understanding of the condition. For these reasons, the establishment of Doctors with ME is to be welcomed. Not only does this constitute a source of support for doctors facing the social, medical and practical problems associated with ME/CFS, but it also creates a group of professionals who are uniquely qualified to comment on the experience of having this illness, especially on the experience of misdiagnosis and lack of understanding on the part of colleagues [14].

This is just one of many recent initiatives which are contributing to advancing the frontiers of knowledge in this area. Others elsewhere in the world include the establishment of the ME/CFS Clinical Coalition in the USA, and in Europe of the EUROMENE (European ME/CFS Research Network) collaboration, which involves more than thirty institutions in twenty-one European countries plus one COST near neighbor country, and means that at last there is in Europe a research infrastructure enabling us to complement the excellent research work being undertaken by our colleagues in North America and elsewhere in the world [15]. As a result, great progress is being made in unravelling underlying pathology, identifying biomarkers, developing better treatments, and discrediting damaging practices. In the UK, the Medical Research Council is at last allocating serious resources to ME/CFS research.

These reports come at a very opportune time. For decades, the study of ME/CFS has been very much a Cinderella specialization within the medical and scientific research community. However, interest has suddenly grown as a result of the increasing phenomenon of Long COVID-19, which has many similarities to ME/CFS. If the current COVID-19 catastrophe has any sort of silver lining, it is that sanity and common sense are beginning to come to the surface of the ME/CFS debate. No one is seriously suggesting that Long COVID-19 is a form of hysteria. Not only is it like ME/CFS, but in many ways it is ME/CFS. Indeed, if there is a silver lining to the current COVID-19 pandemic, it is that the message is finally being understood that long-term neurological and other sequelae following viral infections are genuine clinical entities.

One study found that 89.1% of participants with Long COVID-19 experienced post-exertional malaise, which is generally considered the defining symptom of ME/CFS [16], while a German study found that approximately half of all Long COVID-19 patients fulfilled the diagnostic criteria for ME/CFS [17]. A systemic review by Wong and Weitzer in this issue considered twenty-one studies, and reported much overlap of symptoms between Long COVID-19 and ME/CFS, including fatigue, reduced daily activity, and post-exertional malaise, and some common features in terms of pathophysiology [18]. ME/CFS and Long COVID-19 syndrome have similar symptoms, probably reflecting similar underlying pathological processes involving the central and autonomic nervous system, and a dysregulated immune system. Consequently, a likely consequence of the COVID-19 pandemic will be a considerable increase in the number of people with ME/CFS [19]. Biological abnormalities which appear common to both Long COVID-19 and ME/CFS include redox imbalance, systemic inflammation and neuroinflammation, an impaired ability to generate adenosine triphosphate, and a general hypometabolic state [20]. Such evidence has prompted the proposal of a new taxonomic classification, comprising both Long COVID and ME/CFS within a category entitled post-active phase of infection syndromes, or PAPIS [21].

With regard to the impact of ME/CFS on the quality of life of patients and their families, Brittain et al. [22] conducted a quantitative research study using postal questionnaires. Twenty-four adult volunteers responded, indicating that ME/CFS negatively affects the quality of life of the patient. Additionally, there was a significant correlation between the patient’s reported quality-of-life scores and those of family members. The greatest reduction in the quality of life of ME/CFS patients was in terms of physical health, while that of family members was in terms of worry, family activities, frustration and sadness. Brenna et al. [23] demonstrated that the impact of the illness on net household incomes and quality of life was similar in three different European countries, Italy, Latvia, and the UK. Respondents in all three countries reported similar difficulties in talking to doctors, though differences emerged in patterns and availability of medical and social care, and in societal attitudes. Similar impacts on quality of life have been reported in other contexts, including for example among university students, where stigmatization had a substantial negative impact on subjective self-esteem [24].

There are, not surprisingly, few first-hand accounts by patients with ME/CFS of their illness experiences. However, one seriously affected patient, who fell ill at the age of 21, has recently put his experiences on record. After a year, he was obliged to abandon his wedding photography business because of prolonged post-exertional malaise. He describes his experience of very severe ME/CFS, and reports that he is now bedridden most of the time, and has been unable to leave his room for seven years, except to go to hospital. He has been unable to speak, eat or drink, and has been tube fed. He cannot shower, cut his toe nails, clean his teeth or go to the lavatory, and has had no physical contact with another human being in all that time. It took eight years though, and numerous medical consultations, before he received a diagnosis, but nevertheless he still found himself stigmatized because of lack of knowledge or understanding of the condition. He reports a patient with severe ME/CFS who was forcibly incarcerated in a psychiatric ward and who while there was thrown into a swimming pool to get her to “snap her out of it” and who nearly drowned as a result [25].

Three papers in this Special Issue report on the status of ME/CFS patients in different European countries. In Germany, there may be over 300,000 patients, and Froehlich et al. concluded, on the basis of an online survey completed by 499 respondents who fulfilled the Canadian Consensus Criteria and reported post-exertional malaise, that they were medically underserved. Reported levels of satisfaction with medical care were low, and there were geographical and financial factors limiting the accessibility of medical services [26]. Krumina et al. studied 65 outpatients with fatigue, 55 of whom were diagnosed with ME/CFS. They concluded that patients with more severe ME/CFS were more likely also to have comorbidities, of which fibromyalgia, chronic hepatitis and Lyme disease were of the most frequent occurrences. Fatigue, myalgia, arthralgia and sleep disturbances were more frequently encountered among the ME/CFS patients than among those not diagnosed as having ME/CFS. All the ME/CFS patients reported fatigue and post-exertional malaise. Other symptoms most frequently reported were myalgia, headache, arthralgia and difficulty concentrating. The number of symptoms reported was associated with increased age and longer duration of fatigue. Many of the ME/CFS patients identified physical or mental stress and other chronic diseases as contributing to the development of their illness. Self-help strategies adopted by ME/CFS patients involved physical activities, sleep hygiene, physiotherapy and walking. A total of 90% had taken non-steroidal anti-inflammatory drugs [27]. A comparative survey of ME/CFS patients in Latvia, Italy and the UK found that demographic details were similar in the three countries, as was the impact of the illness on household incomes and quality of life. However, there are differences in illness progression and management, which may be associated with variations in health care patterns and attitudes in society [23].

On the management of ME/CFS in England, the National Institute for Health and Care Excellence (NICE) produced guidance on ME/CFS in 2007 [28]. This proved very unpopular with patients and many professionals, largely because of its espousal of graded exercise therapy (GET) for people with mild or moderate disease [28]. A draft of revised guidance was published in November 2020 which reversed this previous advice, stating ‘Do not offer people with ME/CFS … any therapy based on physical activity or exercise as a treatment or cure for ME/CFS’ [29]. This document was due to be promulgated in its final, definitive form on 18 August 2021, but was withdrawn on the eve of publication because certain elements in the UK medical establishment objected to this change of position [30]. Such a withdrawal of guidance on the eve of publication is unprecedented. There is no provision in the NICE working procedures for this to happen, and it calls into question the much vaunted independence of NICE. GET is simply the immediate casus belli; what is really the issue here is a clash between two opposed points of view as to the nature of ME/CFS, which may be identified as the biomedical and psychosocial paradigms, respectively. NICE has now indicated that a round table meeting, with an independent chairman, will be convened during September 2021, with the intention of bringing the two schools of thought together in order to negotiate an acceptable compromise. However, the chances of success appear slight, as there is absolutely no common ground between the two viewpoints, and in the meantime a letter signed by one hundred and twenty-five of the world’s leading scientific and clinical experts on ME/CFS, including many contributors to this current collection of research papers, has been sent to the Chief Executive of NICE calling for the immediate reinstatement of the withdrawn guidance [31].

If NICE accepts the position of those lobbying for changes to the new guideline on ME/CFS, it will be putting itself at variance with expert opinion in much of the rest of the world. The clinical working group of the European Network on ME/CFS (EUROMENE) has produced guidelines on diagnosis, management and service provision for ME/CFS, and these are published in this Special Issue [32]. They outline impediments to effective clinical care, including unavailability or inaccessibility of services, and unsympathetic attitudes or disbelief on the part of medical practitioners. On diagnosis, EUROMENE recommends use of the Canadian Consensus Criteria [33]. It states that the CDC-1994 (Fukuda) case definition [34] can be used for screening purposes, with the addition of post-exertional malaise as an essential symptom. It goes into considerable detail about the conduct of the consultation and the treatment options available, with the notable omission of graded exercise therapy (GET). In this, the EUROMENE expert consensus is consistent with recent guidance from the US ME/CFS Clinical Coalition, which goes further in stating that GET can worsen the patient’s condition, and that it represents ‘an outdated standard of care’ [35]. It should be noted that, in the paper by Brenna et al. comparing the position of ME/CFS patients in three European countries, only in the UK had respondents received GET, and, of seventy people who had experienced it, only one reported any positive benefit from it [23].

This increasing concern about the use of GET is mirrored in other parts of the world. The Royal Australasian College of Physicians published guidance in 2002 [36]. However, the Australian ME/CFS Advisory Committee reported in 2019 that this guidance has more recently been a cause for concern, partly because of its espousal of graded exercise therapy, and commented: “… the historical context of this guideline must be noted, as they were developed at a time when not much was known about ME/CFS” [37], and stated that, for patients with a significant disability wishing to access care through the National Disability Insurance Scheme, or in receipt of a Disability Support Pension, graded exercise therapy may be inappropriate [38].

A systematic review on the cost-effectiveness of ME/CFS interventions considered ten economic evaluations based in randomized controlled trials, with varying results. It appeared from three studies that cognitive behavior therapy (CBT) may be cost-effective, but this may depend on context, and there was some suggestion from two trials that GET may be cost-effective [39]. However, such equivocal evidence of cost-effectiveness is far from indicating that such treatments may be curative, as their proponents have argued. The socioeconomics working group of EUROMENE drew attention to the costs incurred by those large numbers of patients with ME/CFS whose condition is never diagnosed, often because of the refusal of many doctors to recognize it as a genuine clinical entity [40]. In Latvia, it is thought that the number of undiagnosed patients may be five times greater than those who are diagnosed. It is estimated that the direct medical costs alone for these undiagnosed patients may have been more than €15 million p.a. before the start of the COVID-19 pandemic, and may now be in the region of €17 million [41].

There is general agreement that management of ME/CFS needs to be very much patient centred, and that it is essential for patients to be at the centre of, and very much involved in, clinical decision making in respect of their treatment. In this context, the paper by Ardestani et al., in this collection, is a valuable reminder of the importance of such a person-centred, holistic approach. Their review is of twelve studies of mind–body interventions, including seven randomized controlled trials. The outcomes which they observed were mostly severity of fatigue, anxiety/depression, quality of life, and physical and mental functioning. They found general improvements in these outcome parameters. However, these findings must be regarded as provisional, due to small sample sizes, heterogeneous diagnostic criteria, and the possibility of various biases [42].

To complete the contribution of this Special Issue to the management of patients with ME/CFS, Pheby et al., on behalf of EUROMENE, considered the possible role of preventive programs in minimizing the impact of ME/CFS. They considered in detail the economic case for prevention, as well as the possible health benefits. They concluded that primary prevention would be of little benefit, as not enough is known about modifiable risk factors which could be the subject of such programs. The only exception was in the use of agricultural chemicals, in particular organophosphates. However, secondary prevention is a different matter altogether, and programs to minimize diagnostic delays would have a beneficial effect on both health and the costs of care, by reducing the incidence of prolonged and severe disease [43]. An important element in implementing such a secondary prevention strategy must be measures to address the problems of disbelief and lack of knowledge and understanding among doctors referred to above [10,11,12,13].

In conclusion, the reconsideration in this Special Issue of the 1950s epidemic outbreak of ME/CFS known as Royal Free disease, and effective invalidation of the ‘epidemic hysteria’ hypothesis attached to it, comes at a very opportune time when knowledge of the underlying pathology of the disease is increasing rapidly. Four papers considered the problem of disbelief among many doctors, much of it undoubtedly fuelled by the ‘epidemic hysteria’ hypothesis, and lack of knowledge and understanding of ME/CFS among doctors, with consequent widespread dissatisfaction among patients. In this context, the formation of new organizations specifically concerned with particular aspects of ME/CFS is helpful. Doctors with ME addresses the problems faced by doctors themselves who have the misfortune to have the illness, and who as a result have not only the symptoms of the disease to contend with, but also the opprobrium of colleagues. EUROMENE, the European ME/CFS Research Network, was established to facilitate collaborative working between scientific and clinical colleagues across Europe, and to bring a more strategic approach to the research endeavor. The COVID-19 pandemic has brought with it an upsurge in the number of people experiencing a post-viral syndrome which in many cases is indistinguishable from ME/CFS. This is underlined by an important paper in this collection, and led us to propose a new taxonomic classification category which we are calling post-active phase of infection syndromes, or PAPIS. This Special Issue has also considered in detail the impact of ME/CFS on the lives of patients. One paper considered the quality of life of patients and their families in the UK, while another compared quality of life and other aspects of the illness in three European countries and found many similarities. Other papers reviewed the position of people with ME/CFS in Germany and Latvia, respectively. On management of ME/CFS, the EUROMENE expert consensus document comes at a very opportune moment, when in the UK a last-ditch attempt is being made to subvert and derail the NICE process of guideline development, and to undermine the intention of the draft guideline to abandon NICE’s previous espousal of graded exercise therapy, which has proved very damaging to many patients in the past. This is very unfortunate. The hope is that this attempt will come to naught, as the draft guideline was in line with the growing body of expert opinion throughout the world, including not only the EUROMENE consensus document but also that of the US ME/CFS Clinical Coalition. There is general recognition that the treatment of ME/CFS needs to be very much patient-centred, and this is supported by the paper in this collection on mind–body interventions. Finally, the review of the scope for prevention in ME/CFS underlines once again the need for early detection in order to achieve secondary prevention and minimize the incidence of severe, prolonged disease. The impact of the errors of the past on patients and their families has been immense, particularly on those families of professionals working within the health care system. These are not victimless errors. The consequences can be observed in destroyed reputations, shattered families and family finances, wrecked careers, and blighted lives. However, at long last, there is the prospect of real progress being made through research to unravel the enigma of this very damaging disease. Many of us who have been working in this field for many years have ploughed a difficult furrow, but at last, though the challenges remain immense, the future is looking bright.

## Data Availability

Not applicable.
